# Opportunities for shared decision-making about major surgery with high-risk patients: a multi-method qualitative study

**DOI:** 10.1016/j.bja.2023.03.022

**Published:** 2023-05-02

**Authors:** Sara E. Shaw, Gemma Hughes, Rupert Pearse, Ester Avagliano, James R. Day, Mark E. Edsell, Jennifer A. Edwards, Leslie Everest, Timothy J. Stephens

**Affiliations:** 1 Nuffield Department of Primary Care Health Sciences, University of Oxford, Oxford, UK; 2 Faculty of Medicine & Dentistry, Queen Mary University of London, London, UK; 3 Hammersmith Hospital Imperial College Healthcare NHS Trust London, London, UK; 4 Department of Anaesthesia, Oxford University Hospitals Foundation Trust, Oxford, UK; 5 Department of Anaesthesia, The Royal Brompton & Harefield Hospitals, London, UK; 6 Department of Anaesthesia, Royal Alexandra Hospital, Paisley, UK; 7 Patient Representative, London, UK

**Keywords:** cardiac surgery, colorectal surgery, decision-making consultation, high-risk patients, major surgery, multi-method qualitative study, orthopaedic surgery, shared decision-making

## Abstract

**Background:**

Little is known about the opportunities for shared decision-making when older high-risk patients are offered major surgery. This study examines how, when, and why clinicians and patients can share decision-making about major surgery.

**Methods:**

This was a multi-method qualitative study, combining video recordings of preoperative consultations, interviews, and focus groups (33 patients, 19 relatives, 36 clinicians), with observations and documentary analysis in clinics in five hospitals in the UK undertaking major orthopaedic, colorectal, and/or cardiac surgery.

**Results:**

Three opportunities for shared decision-making about major surgery were identified. Resolution-focused consultations (cardiac/colorectal) resulted in a single agreed preferred option related to a potentially life-threatening problem, with limited opportunities for shared decision-making. Evaluative and deliberative consultations offered more opportunity. The former focused on assessing the likelihood of benefits of surgery for a presenting problem that was not a threat to life for the patient (e.g., orthopaedic consultations) and the latter (largely colorectal) involved discussion of a range of options while also considering significant comorbidities and patient preferences. The extent to which opportunities for shared decision-making were available, and taken up by surgeons, was influenced by the nature of the presenting problem, clinical pathway, and patient trajectory.

**Conclusions:**

Decisions about major surgery were not always shared between patients and doctors. The nature of the presenting problem, comorbidities, clinical pathways, and patient trajectories all informed the type of consultation and opportunities for sharing decision-making. Our findings have implications for clinicians, with shared decision-making about major surgery most feasible when the focus is on life-enhancing treatment.

**Editor's key points**

• Shared decision-making between patients and clinicians about whether to proceed with surgery may lead to high-quality and highly acceptable decisions. However, different types of consultations might provide more or less opportunity to share decision-making.
• This qualitative study combined video recordings and direct observation of preoperative consultations, interviews, and focus groups. Patients and clinicians making decisions about major orthopaedic, colorectal, and cardiac surgery were included.
• The surgical condition, comorbidities, clinical pathways, and patient trajectories all informed the type of consultation and opportunities for sharing decision-making. Evaluative and deliberative consultations offered more scope for shared decision-making than resolution-focused consultations.
• The applicability of these findings to other healthcare systems and surgical specialties remains to be confirmed.

Shared decision-making is a collaborative process: clinicians and patients work together to share information about treatment and management options, consider preferred outcomes and reach agreement on the best care package for the patient.^1^ In the United Kingdom (UK), a landmark legal case in 2015^2^ expedited the shift to shared decision-making, focusing on what a patient would reasonably want or need to know.^3^, ^4^, ^5^ Guidance followed,^6^ along with a shift to patient-centred care.^7^, ^8^, ^9^

Systematic reviews^10^, ^11^, ^12^ show that patients and clinicians value shared decision-making, patients tend to prefer it, and it has the potential to improve the quality of decisions (e.g., via information sharing) and reduce conflict around preference-sensitive treatment decisions about surgery. There is a small but growing body of literature focused on shared decision-making for major surgery with high-risk patients (i.e., older patients and those with severe long-term illness, who are often at high risk of postoperative complications^13^^,^^14^). Even when surgery and anaesthesia are straightforward, one in three high-risk patients develops serious medical complications shortly after surgery.^15^ Many never recover, suffering significant reductions in long-term quality of life and survival.^15^^,^^16^ Some experience regret over the decision to undergo surgery.^17^ Doctors want to improve decision-making for these patients but are often ill equipped to do so.^18^ Clinicians and patients are being asked to talk about decisions, but sometimes lack the knowledge, expertise, or both about how to balance longer-term consequences with the need to address acute problems. High-risk patients often do not realise they have a choice about surgery, and have unrealistic expectations about postoperative recovery.^19^, ^20^, ^21^, ^22^, ^23^, ^24^ There remains a dearth of literature assessing the impact of acuity on decision-making processes, preferences, and outcomes.^25^ Limited attention has been given to communication about surgical decision-making or when and how a decision is shared. In this study, we explore how, when, and why do clinicians and high-risk patients share decision-making about major surgery.

## Methods

**Fig 1:**
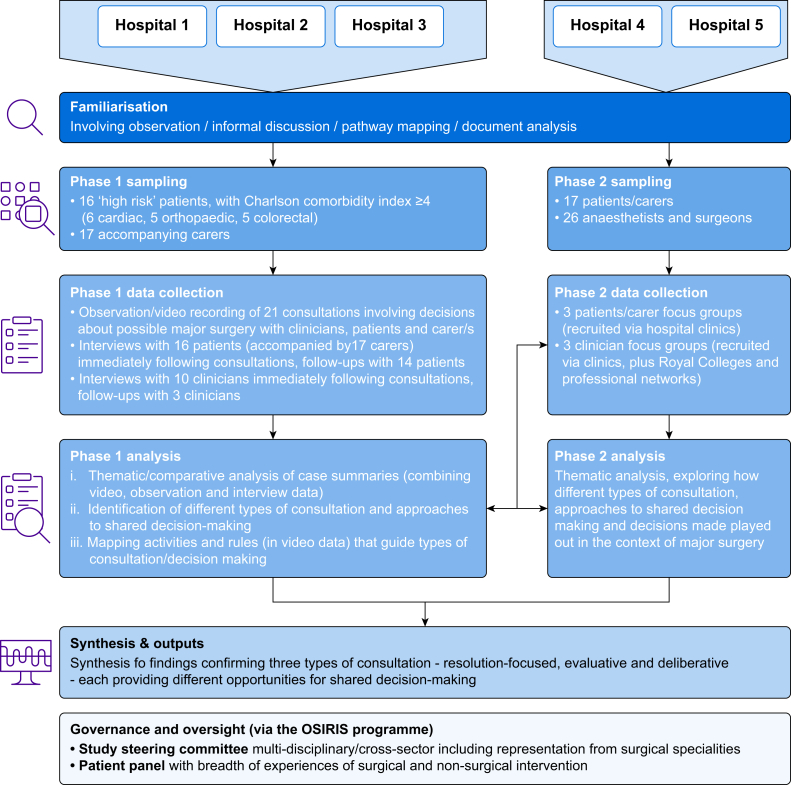
Overview of study approach and methods.

We used multiple qualitative methods, informed by an interpretivist approach. Fig 1 provides an overview. Further detail is available in Supplementary material and the study protocol.^26^

### Ethics and governance

The study received ethical approval from South Central Oxford C Research Ethics Committee (19/SC/0043).

The study is part of the OSIRIS research programme (Optimising Shared decision-makIng for high RIsk major Surgery, https://osiris-programme.org/). The OSIRIS Steering Committee maintained oversight of the research. The OSIRIS Patient Panel informed study design.

### Setting

The research was conducted in five UK National Health Service hospitals (Fig 1) undertaking two of three surgical procedures selected in the OSIRIS programme as three prototypical surgeries representing a spectrum from life-enhancing to life-saving: major orthopaedic, colorectal, and cardiac surgery. Sampling of sites ensured diversity in location, populations served, and hospital size. In an initial familiarisation phase, we mapped clinical settings and pathways.^26^ This informed sampling and later analysis.

### Sampling

We recruited a purposive sample of 33 patients and 19 carers in three sites.

We first identified patients aged 60 yr or older who were considered high risk with a Charlson Comorbidity Index (CCI)^27^ score of ≥4 indicating significant comorbidities or clinically frail (for cardiac surgery, patients' risk was primarily related to their cardiac problem; Table 1). We recruited 16 patients (five orthopaedic, five colorectal, six cardiac), across three sites who were currently undergoing care, ensuring diversity in age, sex, and social circumstances. Fifteen patients were accompanied by at least one carer (*n*=17).

**Table 1:** Summary of patient characteristics, conditions, and decisions. CCI, Charlson Comorbidity Index; CNS, clinical nurse specialist; F, female; GP, general practitioner; M, male; P, patient; PCI, percutaneous coronary intervention.

ID	Site	Patient	CCI	Presenting problem	Consultations observed	Accompanied by	Decision made	Type of decision-making
*Colorectal patients (n=5)*								
P01	1	78 (M)	5	Positive result from bowel cancer screening	1, with consultant surgeon + CNS (Jun 19)	Wife	Major surgery (right hemicolectomy to remove tumour and rejoin bowel)	Resolution-focused
P02	1	74 (M)	5	Anaemic	1, with consultant surgeon + CNS (Jun 19)	Wife	Major surgery (right hemicolectomy to remove tumour and rejoin bowel)	Resolution-focused
P05	2	79 (M)	5	Anaemic, history of stomach cancer	1, with consultant surgeon (Aug 19)	Wife + sister	Transanal minimally invasive surgery to remove part of rectal tumour	Deliberative
P06	2	78 (M)	8	Abnormal colon detected during scan for respiratory problems	1, with consultant surgeon (Sep 19)	Wife	Surveillance (colonoscopy)	Deliberative – shifting to evaluative
P14	2	65 (F)	6	Weight loss, escalating rapidly to pain (previously not disclosed to clinical team)	2, with consultant surgeon (Jan 20) and consultant anaesthetist (Jan 20)	Daughter	Emergency surgery to address obstructing bowel and create stoma	Deliberative
*Orthopaedic patients (n=5)*								
P03	2	76 (F)	5	Pain and mobility problems in hips (bilateral hip replacement 21 yr previously)	3, with consultant surgeon (Aug 19, Oct 19, Dec 19)	Son	Watch and wait follow-up appointments with consultation surgeon	Evaluative
P04	2	75 (M)	4	Knee pain (replacement knee 13 yr previously)	3, with consultant surgeon (Aug 19, Oct 19, Dec 19)	Wife	Surgery to resurface patella –with option to revise joint if required	Evaluative
P07	1	68 (F)	3	Knee pain (replacement knee 5 yr previously)	1, with consultant surgeon (Oct 19)	No	Further investigations/watch and wait	Evaluative
P08	1	70 (M)	4	Knee pain (replacement knee 18 months previously)	1, with consultant surgeon (Oct 19)	Wife	Further investigations/watch and wait	Evaluative
P09	2	81 (F)	5	Knee pain	1, with consultant surgeon (Oct 19)	Friend	Knee replacement	Evaluative
*Cardiac patients (n=6)*								
P10	3	74 (F)	3	Breathlessness and fatigue	1, with consultant surgeon (Nov 19)	Husband	Aortic valve replacement	Resolution focused
P12	1	66 (M)	3	Chest pain	1, with consultant surgeon (Jan 20)	Wife	Coronary artery bypass surgery	Resolution focused
P13	1	77 (M)	3	Aortic aneurysm detected during scan for spinal problems	1, with consultant surgeon (Jan 20)	Wife + daughter	Surgery not advised (owing to calcification of aorta)	Resolution focused
P15	1	72 (M)	3	GP detected ‘murmur’ and referred to cardiologist	1, with consultant surgeon (Jan 20)	Wife	Mitral valve repair	Resolution focused
P16	3	81 (F)	5	Aortic aneurysm detected during investigations for breathlessness, referral to cardiac surgeon had been delayed as multiple myeloma diagnosed in meantime	1, with consultant surgeon (Jan 20)	Husband	Updated imaging required, investigations ongoing	Evaluative (because of lack of information)
P17	3	68 (M)	3	Under care of cardiologist- has heart disease and PCI previously	1, with consultant surgeon (Jan 20)	Wife	Referral to respiratory specialist (now lost to follow-up)	Resolution-focused *but* ended with evaluative as referred on

We recruited a further 17 participants from the remaining two sites for three patient/carer focus groups about past experiences of decision-making about surgery and 26 anaesthetists and surgeons for three clinician focus groups (Fig 1 and Supplementary material). Clinicians were invited to focus groups via Royal Colleges and professional networks. All participants gave written informed consent.

### Data collection

One researcher/co-author (GH) video-recorded 21 consultations (10–45 min) that involved decision-making about major surgery with 16 patients and their carers (June 2019 to January 2020). Two colorectal patients were seen at a ‘hot’ clinic (a ward-based service for those needing urgent treatment) and 14 were seen at outpatient clinics.

The colorectal consultations were planned to discuss options after investigations. Orthopaedic consultations were part of ongoing evaluations. For two patients we recorded three consultations over 5 months. Non-surgical options typically had been ruled out for cardiac patients.

Video-recording involved placing a camera in the consultation room to record interaction between the patient, anyone accompanying them (Table 1), and the clinician(s) (see Supplementary material).

GH conducted narrative interviews^28^ with clinicians and patients (plus carer where relevant) after each consultation, and 5–11 months later. By the end of the study, one patient was still waiting for surgery and one had moved abroad. Another researcher/co-author (TS) and GH facilitated six focus groups (Fig 1).

### Analysis

Analysis was informed by literature on shared decision-making^1^^,^^29^ and the social science of decision-making.^30^, ^31^, ^32^, ^33^

We first used observation data to map clinical pathways and decision-making processes,^26^ and combined this with video and interview data to produce case summaries. Working across case summaries (and returning to the wider dataset as needed), we then used thematic analysis^34^ and constant comparison^35^ to iteratively identify different types of consultation for major surgery and approaches to shared decision-making. Finally, we returned to video data to map activities and examine the substance, form, and rules^33^ of all consultations, conducting detailed analysis of where options were discussed and decisions made. We tested emerging findings in focus groups.

## Results

**Fig 2:**
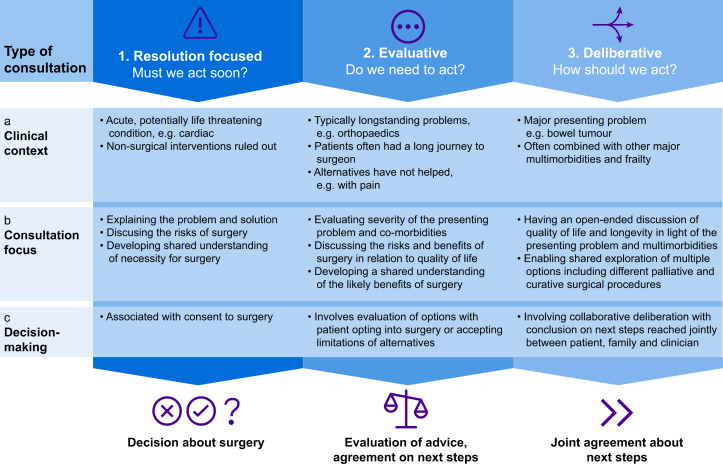
Opportunities for shared decision-making about major surgery.

A significant amount of work took place before patients met with their surgeon to discuss treatment options. This was guided by clinical pathways (e.g., time to reach the surgeon),^26^ patients' eligibility for surgical attention, and multi-disciplinary review. All consultations with the surgeon included discussion about the nature of the problem, causation and prognosis and how it was affecting the patient, explanation of proposed surgery, immediate operative risks (e.g., infection), and what would happen after the consultation and after surgery. Beyond this, analysis (derived from study data) showed that consultations varied, with three types – resolution-focused, evaluative, and deliberative – providing different opportunities for shared decision-making (Fig 2).

### Resolution-focused consultations

These consultations (five cardiac patients, two colorectal patients; Table 1) typically took the form of a traditional consultation, reviewing medical history and explaining the problem/pathology ahead of discussion about treatment. Patients and clinicians understood the problem to be potentially life-threatening, with surgical intervention the optimal route to resolving (or ‘fixing’)^36^ the problem and maximising chances of survival (see Table 2 for examples). In one case, operative risks led the surgeon to recommend against surgery. In another case the consultation became evaluative, with referral for further investigations. Surgeons informed remaining patients about risks and reassured them that surgery was major but routine.

**Table 2:** Data examples for resolution-focused consultations. Drawn from patient and clinician interviews and video-recordings of consultations.

	Resolution-focused consultations
Presenting problem	‘*I had an annual check-up, where they did a blood test. And it was discovered that I was very anaemic* … *which automatically triggers endoscopy and then they found the polyp*’.
	P02, interview
	‘*I feel great! But I get out of breath* … *so breathless and I get tired* … *and when I get short of breath and it tightens all up in my chest*’.
	P10 to cardiac surgeon
Clinician framing of the problem	‘ … *this is a very common cancer* … *most people with it are cured* … *the treatment* … *that we'd recommend would be an operation*’.
	Colorectal surgeon to P01
	‘ … *if we try to operate on this one your chance of stroke will be very high*’.
	Cardiac surgeon, consultation with P13
	‘*I didn't think of it as a decision to be made. When they tell you you've got cancer and we can operate to remove it* … *that was no decision*’.
	Patient with colorectcal cancer, Focus Group 6
Patient understanding	‘*Once I knew that I had the cancer, then it was a matter of coming to terms with that, and getting it sorted as quickly as possible* … *now it's a matter of dealing with what's there*’.
	P01, interview
Discussing choices	‘*We obviously always need to consider the other options but … there's not really any other surgical options … there is the option of doing nothing*’.
	Colorectal surgeon, consultation with P01
	‘*I mean there's an issue here about when I get in a taxi and he asks me which route do you want me to take, and I say,* “*Well hang on a minute, you're the taxi driver*”’.
	Focus Group 1, Cardiac patient
Articulating risk	‘*It's a fairly big operation but we do it routinely and I'll make sure you do well … the overall risk of the operation is about 2% so success is 98%*’.
	Colorectal surgeon, consultation with P10
	‘ … *there are risks involved* … *the risk of surgery is probably about 1% and* … *stroke I think you have a 98 or 99% chance of coming through the operation* … *less serious problems* … *there's a risk of infection* … *chest, urine infection*’.
	Cardiac surgeon, consultation with P15
Reaching a decision	‘ … *in my case he can't do anything* … *you've just got to face the facts*'.
	P13, interview
	‘*really I've got no choice* … *I want a better quality of life* … *although I don't like it* … *I've got a problem* … *get it fixed*
	P15, consultation with cardiac surgeon’.
	‘*It was easy. The pain decided for me*’.
	Focus group 1, orthopaedic patient

These consultations focused on discussing a preferred option, typically surgery. Surgeons had a clear view of the presenting problem and potential benefits of surgery, informed by diagnostics and multi-disciplinary review. Other procedures (e.g., percutaneous coronary intervention for cardiac patients) had already been ruled out. Patients came to the consultation understanding that they had a serious condition that needed fixing (Table 2, presenting problem). Although patients were offered a list of options, (including ‘doing nothing’), there was general agreement that surgery was the optimum choice if the patient was fit enough (Table 2, discussing choices). The content of consultations focused largely on medical knowledge, appreciation of pathology, and weighing up of operative risks (Table 2, articulating risks). Across consultations there was significant discussion of the problem, with images and models used to aid explanation. From the surgeon's perspective, the aim was to help patients understand that surgery was being offered (in one case, not offered), maximise chances of survival, and ensure patients were fully informed and able to consent. Patients in resolution-focused consultations and focus groups, unanimously agreed that this is what they experienced and expected.

In sum, the opportunity for shared decision-making centred around informing the patient about potential surgery and supporting them to make a decision about whether or not to accept it.

### Evaluative consultations

Evaluative consultations involved five orthopaedic patients, plus one colorectal and one cardiac patient. Unlike resolution-focused consultations, there was no predetermined solution. The focus was on evaluating patients' situations and assessing options and benefits (Table 3). Before the surgical consultation, orthopaedic patients had consulted one or more health professionals and received non-surgical interventions (e.g., physiotherapy). Four presented with pain related to a previously replaced joint. All were hoping for resolution. One colorectal and one cardiac patient fell into this category owing to information about comorbidities that became apparent during the consultation.

**Table 3:** Data examples for evaluative consultations. Drawn from patient and clinician interviews and video-recordings of consultations.

	Evaluative consultation
Presenting problem	‘*I'm having problems getting up the stairs* … *there's always been pain*’.
	P03 interview
	‘*I can definitely feel it starting from sort of here coming down* … *particularly if I am going downhill or uphill or upstairs or downstairs*’.
	P04, consultation with orthopaedic surgeon
	‘ … *with the pain I'm getting* … *It stops me in my tracks if I'm doing something*’.
	P07, interview
Clinician framing of the problem	‘ … *your knee is now not functioning quite so well but it's ok* … *you are functioning pretty well*’.
	Orthopaedic surgeon, consultation with P07
	**‘***It's never an operation that you have to have done it's not life-saving. It's meant to improve things for you* … *but there is a possibility that it could make things worse*’.
	Orthopaedic surgeon, consultation with P09
Patient understanding	‘*I can't explain how excruciating it is* … *but it's only for a short period* … *…but I mean if it doesn't get any worse I can tolerate that*’.
	P07, to orthopaedic surgeon
Discussing choices	‘ … *so that's the plan to re-surface the knee cap and or revise the whole knee that's one side* … *the second one is wait and watch* … *here's two approaches to this* … ’.
	Orthopaedic surgeon consultation with P04
	‘*It's your decision* … *I'll help you try to get to what you feel is the right thing* … *but it's there is quite a lot of uncertainty so you have to have a bigger operation with a longer recovery where there's a risk of* … *complication blood clot, infection, problems with your heart and your lungs these sort of things*'.
	Orthopaedic surgeon, consultation with P08
Articulating risk	‘*So the main risks that you need to know about are infection* … *so that's about 1% probably slightly higher because you're on the warfarin* … *despite the fact that you'll be on your warfarin, there is still that risk of blood clots* … *the other one the other big one is ongoing pain and stiffness so it might not give you the result that you want from it* … *there are some other ones that are more minor*’.
	Orthopaedic surgeon, consultation with P09
Reaching a decision	‘*I wouldn't mind going for the* [surgery]’.
	P04, consultation with orthopaedic surgeon
	‘*I was hoping it wouldn't come to that but at the same time if its needs done and its going give me less pain and a bit more mobility I would do it*’.
	P09, consultation with orthopaedic surgeon

From the surgeon's perspective these were consultations about *life-enhancing*, rather than *life-saving*, treatments. Surgeons focused on evaluating if surgery was likely to help, or if frail and multi-morbid patients could be worse off as a result. Decision-making focused on what was best for the patient. For all orthopaedic consultations the surgeon's knowledge guided encounters: surgeons typically clarified pathology and the likelihood of surgery helping, summarised the extent of problems and associated pain, weighed up potential risks and benefits, and cautioned about the likely success of surgery. Potential risks and outcomes for treatment options were frequently (but not always; Table 3) quantified. Examination, imaging, and models were used to assess and explain the surgical condition and treatment options.

Two orthopaedic patients decided that surgery could be of benefit; three decided against surgery and were offered follow-up appointments and advice (Table 1). The colorectal and cardiac patients were referred for further diagnostic tests. Decisions against surgery were framed as ‘watch and wait’ decisions, keeping options open.

In sum, opportunities for shared decision-making involved developing a shared understanding of potential benefits of surgery, led by the clinician and often involving discussions over several consultations. Potential benefits/risks (in terms of surgery and patients' potential quality of life improvements) were evaluated relative to comorbidities. Patients were supported to make a decision: accepting surgery or continuing with non-surgical management.

### Deliberative consultations

Deliberative consultations involved three colorectal patients. In these consultations, it was the high-risk status of the patient combined with their presenting problem – here, colorectal tumours – that was paramount when considering next steps (see examples in Table 4). Unlike resolution-focused and evaluative consultations, discussion about the potential benefits of surgery was explicitly linked with patients' frailty and likely consequences (e.g., hospitalisation), with alternative surgical and non-surgical options considered alongside discussion about anaesthetic procedures. This opened up more opportunities for collaborative construction of options, and shared decision-making, than other consultation types.

**Table 4:** Data examples for deliberative consultations. Drawn from patient and clinician interviews and video-recordings of consultations.

	Deliberative consultation
Presenting problem	‘*I went to my doctor* … *and he put me on iron tablets because* … *and he said,* “*I'm going to send you for a scope both up and down* …” *and that's where it started. He said,* “*They've obviously spotted something*”’.
	P05 during interview
	‘ … *pain* … *vomit* … *we've realised* … *the gut is quite different from when it was when we saw you*’.
	Surgeon to P14
Clinician framing of the problem	‘*He tried to convince me last time that he was really fit and well* … *there was some warning bells* … *An early rectal cancer* … *opens up the option* … *we were talking about*’.
	Colorectal surgeon (about P05), interview
	‘*So, I start with a similar sentence* … “*Do you know why you've come to see me?*” *and I just stay quiet, and most of them go,* “*Well I'm not really very fit, am I?*” *To begin with that pause, they'll fill it in and you're on the right page with them* … ’
	Anaesthetist, focus Group 1
Patient understanding	‘*Well I was told there were 3 options*’*.*
	P05, consultation with colorectal surgeon
	‘*I didn't know they were going to cut as much away*’.
	P06, interview
Discussing choices	‘*Do you want the major surgery* … *or the* [local surgery]? *It's not something that's offered to everyone but your results so far are going to suggest that's an option*’.
	Colorectal surgeon, consultation with P05
	‘*If we chose nothing* [we'd] … *have a chat with the palliative care doctors and see if we manage that just as comfortably as we can for you … but it's not going to treat it and it's not going to take anything away* … *you've got a couple of surgical options so they all involve a general anaesthetic*’.
	Colorectal surgeon, consultation with P14
Articulating risk	‘*It's explaining what risk is. You can say someone's high risk but you have to say high risk and all the complications; higher risk of a longer hospital stay, higher risk of not getting back to your current function capacity, needing carers*’.
	Focus Group 1, anaesthetist
Reaching a decision	‘*I personally feel that's a better option if you could handle it, but it's you that's got to go for the decision.* [yes] *I'll go for that then*’.
	P06 wife and P06 during consultation
	‘*You have ruled out the* “*no*” *I think haven't you? And you're kind of ruling out, I think, the more extreme longer version* … *you're in the middle*’.
	Colorectal surgeon, consultation with P14

Collaborative deliberation^31^ of options was a key feature of deliberative consultations and focused on what survival might mean in the context of patients' clinical, social, and family situation (e.g., need for long-term carer support postoperatively). This deliberative aspect stood out in comparison with other types of consultation. One surgeon led deliberative evaluations with three patients with colorectal tumours, for which surgery would usually be warranted but in these cases was questioned given comorbidities. Anaesthetists and colorectal surgeons in focus groups stressed the value of this approach for higher risk patients while acknowledging the challenges, including the time needed for discussion.

Consultations were framed in terms of what mattered to patients (e.g., discussing choices; Table 4). Longer-term risks of harm, reduced quality of life and level of uncertainty were explicitly discussed, allowing patients, relatives, and clinicians to weigh up risks, benefits, and uncertainties (e.g., articulating risk; Table 3). Operative risk was raised, including risk of mortality, likelihood of needing long-term care, and potential to live independently after surgery.

Options were explored collaboratively. Patients were asked to make their decision from a list of options. Two patients opted for lesser (one palliative, one less invasive) interventions. One consultation shifted into an evaluative discussion about how much the tumour was a problem in light of a respiratory problem, leading to a decision to ‘watch and wait’.

In sum, these consultations provided more opportunity for shared decision-making. Although the choice of treatment was down to the patient, the high-risk status of the patient framed discussion and enabled collaborative (i.e., shared) deliberation about risks, uncertainties, and potential benefits.

## Discussion

This study has shown that, in contrast to current UK guidance, treatment decisions about major surgery for patients at high risk of poor long-term outcomes are not necessarily shared decisions. The combination of qualitative methods and sensitivity to the processes of decision-making involving high-risk patients allowed us to: (1) reveal how surgeons adopt distinct and varied approaches to consulting with patients about major surgery; (2) identify three types of consultation, shaped by variable clinical contexts, that offer different opportunities for shared decision-making; (3) raise the possibility that shared decision-making is not always possible or desirable; and (4) highlight how, although decisions are made at specific time points and in dedicated decision-making consultations (typically a significant way along a clinical pathway), in practice they unfold over time and across multiple encounters. Our findings add to the literature on shared decision-making with high-risk patients, which indicates that discussions between surgeons and patients about potential postoperative complications often have significant communication gaps,^19^^,^^20^ with reliance on surgical expertise and experience over individual, preference-sensitive choice. Both parties tend to *assume* shared values, which shapes decision-making (e.g., patients lack of belief in the surgeon's prognosis informs their decision).^21^

Previous studies suggest a mis-match between clinician and patient preferences for participation in decision-making^23^^,^^37^^,^^38^ and between what surgeons discuss and what patients want to know (typically less technical information, and more on survival and longer-term quality of life).^39^ Such studies rarely distinguish between different types of consultation. Our findings show that options in resolution-focused (or ‘fix it’)^36^ consultations are perceived to be extremely limited (e.g., surgery or death) or non-existent. This does not mean that resolution-focused consultations are not patient-centred, but that they focus more on creating a shared understanding of surgery. Patients in these consultations wanted to have their problem fixed, saw that as the surgeons' role and (whether or not they had surgery) accepted both process and decision made. As reported elsewhere, care is needed to avoid focusing on ‘fixing’ a problem in ways that close down discussions^36^; and avoid allowing ‘clinical momentum’^1^^,^^22^^,^^36^ to guide judgements about patients' disposition for shared decision-making.^19^

Our findings suggest that shared decision-making is not necessarily possible or desirable in resolution-focused consultations. Evaluative and deliberative approaches involve discussion of options and the consequences of those options in the context of each patient's situation and preferences to generate a shared understanding, which guides the final decision. This aligns more closely with conceptual models of shared decision-making.^31^^,^^40^ Clinical and patient context is key to appreciating these two consultation types, with evaluative consultations involving significant periods of time and often complex pathways to decision points about life-enhancing treatment, and deliberative consultations foregrounding the high-risk status of the patient as critical to decision-making alongside the presenting problem (Table 3).

Our findings have potential to integrate with current shared decision-making models, including the Three-Talk model,^41^ underpinned by Collaborative Deliberation^31^ and Mind-It^42^ models, which propose an opening up of patient–clinician communication such that a shared understanding of the problem, possible treatment options, and patients' values and goals can be generated, leading into deliberation and shared decision-making. None of these models currently focus on surgical decisions, the needs of high-risk patients, or the varied extents to which shared decision-making may be feasible or desirable in different types of consultation. There is a need for greater appreciation of how resolution-focussed consultations have far fewer opportunities for shared decision-making, and how evaluative and deliberative consultations open up opportunities that are far more likely to lead to shared understanding, and shared decision-making. Acknowledging this alongside other models would create explicit awareness of how shared decision-making can work differently in different types of consultations and involve the patient to the fullest extent possible in each. We propose training that enables awareness of consultation type, alongside standardised decision support.

### Strengths and limitations

As is frequently the case in qualitative research, our patient sample was small. We ensured breadth of sampling and used multiple methods to generate a rich dataset enabling in-depth analysis of consultations. Interactional data combined with interviews provided detailed insights on the process of decision-making, allowing us to identify when and how decisions were made and the extent to which they were shared. Testing emerging analysis with a wider group of clinicians and patients in focus groups was helpful, albeit limited in terms of clinical speciality (involving neither orthopaedic nor cardiac surgeons, two of the three surgical specialities in the study) and patient experience. Further research is needed to appreciate the extent to which different consultation types play out by speciality, and the potential for a range of factors (not measured in our study) such as clinician and patient activation, health literacy, and ethnicity to influence shared decision-making.

We used the CCI^27^ to help identify high-risk patients. Some patients with lower scores were considered by clinicians to be high risk and *vice versa*. We sought to address this by working with recruiting clinicians to include patients identified as high risk. It is possible that the same study conducted in different sites would identify different types of high-risk patients. The five hospital sites in the study varied in location, populations served, and size; however, there remain limits on the transferability of findings to other jurisdictions and healthcare settings.

### Conclusions

The dominant assumption in healthcare is that shared decision-making is relevant to every consultation. This study showed that the traditional medical consultation was reinforced in resolution-focused consultations with limited focus on shared decision-making (which may be appropriate for some patients). Evaluative and deliberative consultations provided greater opportunities for shared evaluation of the potential benefits of surgery in specific types of consultation. Deliberative consultations were more appropriate for older, frail patients for whom the longer-term outcomes of surgery were uncertain. Training that can support different approaches to shared decision-making across these different types of consultation is urgently needed.

## Author's contributions

Original study design: SS, RP, TS, LE

Principal Investigator for the study and guarantor for the article: SS

Participant recruitment across study sites: EA, JD, ME, JE

Collected data for interviews and video-recording of consultations: GH

Collected data for focus groups: TS, SS, GH

Analysis and interpretation of data: SS, GH

Analysis of focus group data: TS

Drafting of the article: SS

Revision of the article: SS, GH, TS, RP

All authors commented on a draft version of this paper, approved the final version and agreed to be accountable for all aspects of the work.

## Declarations of interest

RP has received research grants, honoraria, or both from Edwards Lifesciences, Intersurgical and GloaxoSmithkline, and is a member of the editorial board of the *British Journal of Anaesthesia*.

## Funding

The study reported here forms the first part of the OSIRIS research programme (Optimising Shared decision-makIng for high RIsk major Surgery, https://osiris-programme.org/), funded by the UK National Institute of Health Research (RP-PG-0217-10001). The views expressed are those of the authors and not necessarily those of the NIHR or the Department of Health and Social Care.
