# Supplementary material for: Opportunities for shared decision-making about major surgery with high-risk patients: a multi-method qualitative study
Source: Br J Anaesth. 2023 May 2;131(1):56–66. doi: 10.1016/j.bja.2023.03.022 (PMC10308437; doi:10.1016/j.bja.2023.03.022)
Supplement: Multimedia component 1 [file mmc1.docx]

**Supplementary Material – additional detail about study design, approach and methods**

This supplementary document provides further detail on the study design and approach and methods used in the study presented in the main paper. In particular it includes further information relating to the rational for the methods used, analytic objectives and analytic process. Readers can also refer to the original study protocol should they wish – though this is not necessary to be able to appreciate the methods and approach used.

This is not intended as a standalone document, but should be read in conjunction with the main paper where information about the research programme, ethics approval, background to the study, results and discussions – as well as summary methods and overview figure - can all be found.

**Overview of study design and approach**

Shared decision making aims to bring together clinician expertise and patient values and preferences in order to decide on the best package of care for the patient. In this study we therefore used multiple qualitative methods to explore in-depth how patients, their families and clinicians negotiate decision making and reflect back on the decisions they made.

The study involved a multi-disciplinary team, bringing representation from medicine, nursing, sociology and social policy. We drew on practice theory to situate the study and focus our attention on decision-making-in action (Shaw, Hughes, Stephens et al, 2020). This came from a recognition that decisions to have (or not to have) surgery are rarely made at neat ‘decision points’ and are instead distributed over time and space (Rapley 2008), involve varying degrees of interaction and deliberation and are shaped, enabled and (potentially) constrained by a wide range actors and artefacts (Elwyn, Lloyd, May et al. 2014; Feldman & Orlikowski 2011).

In terms of study design and approach, this led us to adopt an interpretivist approach that situated the process of decision-making (and activities allied to it, e.g. consultations, family discussion, accessing patient leaflets) as something that happens through an on-going process of communication, interaction and collaborative articulation of what major surgery might mean for those involved. Our focus was on understanding how meanings about surgery get constructed and how communication and interaction unfold, particularly (but not only) in consultations where decision making is paramount.

**Additional information about study methods**

*Sampling and data collection*

We provide an overview of sampling and methods of data collection in the main paper, and a detailed summary in Table A below.

The research was conducted in five NHS hospitals, ensuring maximum diversity in location, populations served and hospital size. All five hospitals were undertaking two of three surgical procedures selected in the OSIRIS programme as three prototypical surgeries representing a spectrum from life-enhancing to life-saving – major joint, colorectal & cardiac surgery. The rationale for this in the main OSIRIS programme was that: (a) major joint replacement for osteoarthritis is a symptomatic treatment which will not prolong life but can improve quality of life for those with significant pain and reduced mobility - it is likely to be considered and discussed within primary care, as well as specialist musculoskeletal services, as part of a potentially long-term process of considering surgery with an orthopaedic team; (b) colorectal surgery for bowel cancer is essential and requires relatively rapid decisions about treatment - following diagnosis patients, relatives and clinicians are faced with choices about the nature of the procedure (including a potential for palliative surgery) and the need for adjunct radiotherapy or chemotherapy, and (c) coronary artery bypass grafting may prolong life at a population level, but for the individual patient this benefit is not guaranteed, especially for frailer or multimorbid patients - increasingly, less invasive, percutaneous coronary interventions have created a range of options for patients with ischaemic heart disease.

Within site sampling and data collection was conducted in three main phases: (a) an initial familiarisation phase involving initial observation, informal discussion with clinical teams across the three specialities (see above), document analysis and mapping of clinical pathways; (b) video-recording of decision making encounters (typically consultations with surgeons and patients, and family/carers), and (c) follow up interviews with patients and clinicians having either had or declined surgery, as well as focus groups with a wider group of patients, carers and clinicians. We focused on a purposive sample of three of the five hospital sites for video—recording of consultations (Phase 2) and follow up interviews (Phase 3); and the remaining two sites (along with professional networks) for focus groups (Phase 3).

Table A provides a breakdown of data collected, and analytic objectives for collection of data in each phase of the study. Decision making maps developed in the familiarisation phase for each of the clinical specialities are available in the published study protocol (Shaw, Hughes, Stephens et al, 2020).

**Table A: Overview of data collection and analysis**

| **Focus** | **Data collected** | **Analytic objectives** |
| --- | --- | --- |
| **Familiarisation**: clinical setting and pathways | 16 visits across a maximum variation sample of five NHS hospital sites (totalling 67 hours of observation/100 pages of researcher field notes), including:   - 6 informal meetings with surgeons and other clinicians, - observation of 7 x multidisciplinary team meetings (4 x colorectal, 3 x cardiac, 1 x specialist high-risk anaesthetic multi-disciplinary team meeting) - observation of 3 specialist pre-operative assessment clinics - observation of training on shared decision-making - 21 documents (e.g. patient information leaflets, pathway documents) | Mapping clinical pathways and workflows, and decision points about surgery, informing creation of ‘maps’ (Shaw, Hughes, Stephens et al, 2020)  Understanding team dynamics and interactions about surgical options  Appreciating the information available for patients to inform decision-making  Examining the rationale for doing shared decision-making, when/how and this takes place |
| **Phase 1**: real time decision-making about surgery | Video-recordings of 21 decision-making consultations (totalling 7 hours and 25 minutes of data), in three of hospital sites (with diversity in location, population and size of hospital), involving a purposive sample of 16 high-risk patients and their carers (n = 17)   - 6 with 3 surgeons about cardiac surgery - 5 with 2 surgeons about colorectal surgery - 9 with 3 surgeons about orthopaedic surgery (2 patients each had 3 consultations) - 1 with consultant anaesthetist for colorectal surgery   20 of these consultations included family members/friends | Gaining detailed insights about the ways in which deliberation and interaction (verbal and non-verbal) shapes decision-making; who is involved and how; what artefacts (e.g. leaflets, visual/decision aids) feature and why; and the substance, form and rules of each consultation.  Understanding approaches to decision-making and connections to wider clinical, organisational, social and biographical contexts |
| **Phase 2:** reflections on decision-making about major surgery | 43 audio-recorded interviews at two points: 26 immediately after a decision had been made, and 17 5-11 months later (totalling 16 hours, 4 minutes of audio data), using a topic guide and narrative approach to accessing patient’s accounts of their condition, situation an decision making over time, and involving:   - 16 patients (total of 30 interviews), accompanied by 17 family/friends - 8 surgeons (3 cardiac, 2 colorectal, 3 orthopaedic) (total of 14 interviews covering all 16 patients) - 1 CNS (total of 2 interviews) - 1 anaesthetist (total of 1 interview)   3 patient/carer focus groups, involving 17 participants from two hospitals:   - 5 cardiac, 4 colorectal, and 6 orthopaedic patients; 2 relatives/carers   3 clinician focus groups, involving 26 participants:   - 6 colorectal surgeons, 20 anaesthetists | Gaining insights in to experiences of, and perspectives on decision-making, including hopes and expectations of patients and families, as well as potential regret over time  Gauging reflections of whether decisions are ‘shared’ and what this means in the context of different kinds of surgery and different patient/illness trajectories |

For video-recording of consultations in Phase 2, we worked with clinical teams in each of the three sites to recruit a maximum variation, purposive sample of 16 high-risk patients aged ≥60 years and with an age-adjusted Charlson comorbidity score (Charlson, Szatrowski, Peterson et al, 1994) of ≥4, who were contemplating elective surgery, ensuring variation in age, gender and social circumstances (see Table 1, main paper). We video-recorded consultations in order to capture verbal and non-verbal (e.g. use of computer or decision aids) interaction, and enabling insight into the decision making process as it happened in real time, including content of each consultation (e.g. information exchanged, options discussed) and the interaction (e.g. between clinician and patient, patient and family member). This involved the researcher (Gemma Hughes) placing a video camera in the consultation room and recording the consultation. Where the patient agreed, and it was logistically possible, GH remained in the room. This is usual in qualitative studies, with the researcher’s presence enabling appreciation of each consultation as it unfolds in real time and the video recording facilitating detailed analysis of interaction that is not feasible through observation alone. On occasions where patients preferred not to have the researcher present, or the researcher was interviewing another patient at the time of the consultation, the researcher started the camera then left the room and left it running during the consultation, returning to stop and retrieve the film after the consultation ended.

For interviews, we followed up with patients and clinicians (and carers where relevant) at two points (Table A), as soon as practically possible after their consultation, and 5-11 months later. We adopted a narrative approach [Greenhalgh, Russell & Swinglehurst, 2005) to interviewing, encouraging participants to recount the details of their experiences (e.g., their experience of illness/their condition, accessing care, decision making about surgery, family support). Interviews lasted up to 1 hour and (given the timing of the later part of the study during the COVID-19 pandemic) were either online via Teams or Zoom, or in-person and recorded and transcribed with consent.

For patient focus groups, we worked with the remaining two hospital sites to invite patients who had been offered major surgery across any of the three specialities of interest in the 3-6 months previously (the aim here was to recruit those who were able to reflect back on the decision made, rather than speaking with those – as in interviews – who had recently been involved in decision-making). Patients were initially approached by the relevant clinical team and then contacted by the researcher (Tim Stephens, TS) if they and/or their carer or family member were interested to participate. This resulted in three focus groups involving a total of 17 patients (see Table A for breakdown by speciality). Focus groups were in-person, typically lasted 60-90 minutes and involved broad discussion around experiences of being a high-risk patient and of decision making for major surgery, combined with discussion of decision making scenarios informed by emerging analysis about different consultation types and opportunities for shared decision making with each.

For clinician focus groups, we initially aimed to recruit clinicians from across all three specialities and including clinical nurse specialists. This proved challenging, initially in terms of available time, and then later on in the study in terms of the context of the COVID-19 pandemic, increased workload and requirements for physical distancing. We therefore focused recruitment to a smaller set of specialists and combined recruitment via three key routes: the two remaining hospital sites, Royal Colleges of Surgeons and Anaesthetists and professional networks. Where possible we aimed to piggyback existing events at which clinicians were already participating (e.g. RCA conference). This led to three focus groups, involving 26 clinicians (see Table A for breakdown). Focus groups typically lasted 45 to 60 minutes and focused largely (but not exclusively) on discussion of decision making scenarios informed by emerging analysis about different consultation types and opportunities for shared decision making with each

*Analysis*

Data collected across the three phases provided a rich dataset that allowed us to understand the clinical pathways and workflows guiding decision making about surgery; the interactions, content and flow of real time decision making in consultations; and post hoc reflections about decisions that were made about major surgery – see Table A for a summary of analytic objectives relating to different phases of the study.

In terms of analysis, we first mapped out clinical pathways and decision-making points and processes (see study protocol for detailed maps - Shaw, Hughes, Stephens et al, 2020). We then combined this with interview data and video-recordings to make narrative case summaries, examined similarities and differences across these, and then used this to inform development of decision-making scenarios for high-risk patients considering major surgery. We used thematic (Miles, Huberman & Saldana, 2014) and comparative (Glaser & Strauss, 1967) analysis to identify different types of consultation for major surgery and approach to shared decision-making.

As set out in the main paper, our analysis was informed by literature on shared decision-making and the social science of decision-making. Given our focus on interaction and communication as shaping decision-making-in-action (see above), we drew particularly on work on genres of communication (Yates & Orlikowski, 1992) to examine the interactional order of consultation. This involved: (i) mapping the activities that patients, family and clinicians carried out during each consultation, and the order in which these were addressed as the consultation unfolded, (ii) identifying the substance, form and rules (implicit/explicit expectations of patients and clinicians) for each consultation, and (iii) conducting detailed analysis of interactions where options were discussed and decisions made. This led us to categorise consultations into three distinct types - resolution-focused, evaluative and deliberative – each with different features and prospects for shared decision-making (see Results – main paper).

In practice our analysis began as we collected data and was on-going throughout the study. This meant that we were able to develop a typology of consultations from video and early interview data, which we were able to take to focus groups to explore with participants providing a sense check, with both clinicians and patients, on our emerging analysis.

**Supporting references for supplementary material**

Charlson M, Szatrowski TP, Peterson J, Gold J. Validation of a combined comorbidity index. *Journal of Clinical Epidemiology.* 1994;47:1245-1251.

Elwyn G, Lloyd A, May C, et al. Collaborative deliberation: a model for patient care. Patient Educ Couns 2014;97:158–64. 38

Feldman MS, Orlikowski WJ. Theorizing practice and practicing theory. Organization Sci 2011;22:1240–53

Glaser B, Strauss AL. *The discovery of grounded theory: Strategies for qualitative research*. Aldine de Gruyter; 1967.

Greenhalgh T, Russell J, Swinglehurst D. Narrative methods in quality improvement research. Qual Saf Health Care 2005;14:443–9.

Miles M, Huberman M, Saldana J. *Qualitative Data Analysis: A Methods Sourcebook*. Sage; 2014.

Rapley T. Distributed decision making: the anatomy of decisions-inaction. *Sociology of Health & Illness* 2008;30:429–44. 37

Shaw SE, Hughes G, Stephens T, Pearse R, Prowle J, Ashcroft RE, Avangliana E, Day J, Edsell M, Edwards J & Everest L. Understanding decsion making about major surgery protocol for a qualitatve study of share decsion making by high-risk patients and their clinical teams. *BMJ Open*, 2020, 10:e033703. doi:10.1136/bmjopen-2019-033703.

Yates J, Orlikowski WJ. Genres of organizational communication: a structurational approach to studying communication and media. *The Academcy of Management Review.* 1992;17(2):299-326.
